# Label-Free Monitoring of Uptake and Toxicity of Endoprosthetic Wear Particles in Human Cell Cultures

**DOI:** 10.3390/ijms19113486

**Published:** 2018-11-06

**Authors:** Anika Jonitz-Heincke, Jenny Tillmann, Melanie Ostermann, Armin Springer, Rainer Bader, Paul Johan Høl, Mihaela R. Cimpan

**Affiliations:** 1 Department of Orthopedics, Biomechanics and Implant Technology Research Laboratory, Rostock University Medical Center, Doberaner Strasse 142, 18057 Rostock, Germany; jenny.tillmann@gmx.net (J.T.); rainer.bader@med.uni-rostock.de (R.B.); 2 Biomaterials—Department of Clinical Dentistry, Faculty of Medicine, University of Bergen, Aarstadveien 19-21, 5009 Bergen, Norway; melanie.ostermann@uib.no (M.O.); mihaela.cimpan@uib.no (M.R.C.); 3 Medical Biology and Electron Microscopy Center, Rostock University Medical Center, Strempelstrasse 14, 18057 Rostock, Germany; armin.springer@med.uni-rostock.de; 4 Department of Clinical Medicine, University of Bergen, Jonas Lies vei 87, N-5021 Bergen, Norway; paul.hol@uib.no; 5 Department of Orthopaedic Surgery, Biomatlab, Haukeland University Hospital, Jonas Lies vei 87, N-5021 Bergen, Norway

**Keywords:** wear particles, CoCr28Mo6, alumina matrix composite, impedance-based monitoring, high-resolution darkfield microscopy, field emission scanning electron microscopy

## Abstract

The evaluation of the biological effects of endoprosthetic wear particles on cells in vitro relies on a variety of test assays. However, most of these methods are susceptible to particle-induced interferences; therefore, label-free testing approaches emerge as more reliable alternatives. In this study, impedance-based real-time monitoring of cellular viability and metabolic activity were performed following exposure to metallic and ceramic wear particles. Moreover, label-free imaging of particle-exposed cells was done by high-resolution darkfield microscopy (HR-ODM) and field emission scanning electron microscopy (FESEM). The isolated human fibroblasts were exposed to CoCr28Mo6 and alumina matrix composite (AMC) ceramic particles. HR-ODM and FESEM revealed ingested particles. For impedance measurements, cells were seeded on gold-plated microelectrodes. Cellular behavior was monitored over a period of 48 h. CoCr28Mo6 and AMC particle exposure affected cell viability in a concentration-dependent manner, i.e., 0.01 mg/mL particle solutions led to small changes in cell viability, while 0.05 mg/mL resulted in a significant reduction of viability. The effects were more pronounced after exposure to CoCr28Mo6 particles. The results were in line with light and darkfield microcopy observations indicating that the chosen methods are valuable tools to assess cytotoxicity and cellular behavior following exposure to endoprosthetic wear particles.

## 1. Introduction

Revision of total joint replacements is still a major clinical problem in orthopedic surgery. Aseptic implant loosening is the main reason for revision [1], and is associated with the generation of wear particles and ions. Nowadays, manufacturing processes of endoprosthetic materials are refined to decrease the volumetric wear of the bearing surfaces [2], but the occurrence of wear debris caused by articulation or mechanical instability is still an issue [3]. In periprosthetic tissue, the interaction of different cell types with generated particles as well as ions triggers different cellular reactions [4,5]. Due to the suppression of osteoblastic differentiation and function, and the concomitant induction of osteoclastogenesis, bone degradation processes and inflammation are resulting in osteolysis. The biological reactivity of wear particles depends on: (i) the particle’s physicochemical characteristics, such as size, shape, composition, z-potential; (ii) particle number (dose) and length of exposure, and (iii) host characteristics [4]. To elucidate the mechanisms involved in wear particle-induced osteolysis, the influence of physico-chemical characteristics on uptake into relevant cells and on putative toxic effects has to be determined. The monocytic/macrophage lineage is the major cell type for inflammatory mediator release resulting from their particle-phagocytic role [6,7]. Particles should have a size of not more than 10 µm [8,9,10] to undergo pinocytosis, endocytosis, or phagocytosis, thereby further mediating the pro-inflammatory response. Cellular activation differs depending on the type of foreign body. The soluble ionic form triggers the biological response after phagocytosis or ion-channel-mediated entry, whereas non-phagocytosable complexes mediate the cell response by binding on membraneous toll-like receptors [10,11,12,13,14]. After activation, macrophages release a series of inflammatory cytokines which induce osteoclastogenesis and attract and recruit other cells (macrophages, osteoclasts, fibroblasts) to the periprosthetic site to boost the inflammatory response [4,15]. Additionally, osteoblasts and fibroblasts can phagocytise wear particles [15,16,17] which considerably increase the expression of relevant cytokines and chemokines [15,18,19].

To evaluate the biological effects and toxicity of endoprosthetic wear particles on relevant cell types in vitro, a broad range of test assays and detection systems are commonly used. Since traditional toxicological methods have, in some cases, proven to be susceptible to nanoparticle-induced interferences, the need for label-free testing approaches increases [20]. Therefore, the aim of the present study was to assess the cytotoxicity of selected metallic and ceramic wear particles by label-free darkfield and field emission scanning electron microscopy (FESEM), as well as by label-free live impedance-based monitoring. The main advantage for the latter is the real-time evaluation of the biokinetic behavior of cells and their interaction with toxicants over a period of time [20]. In impedance-based assays, information of cellular properties like proliferation, growth, or adhesion can be generated as a function of signal frequency by applying an alternating current to electrodes which cover the bottom of cell culture plates. Cells growing on the surface of these electrodes impede the flow of the electric current, resulting in current changes which can be measured [20]. In a previous study of Cimpan et al. (2013) [20], this methodological approach was used to assess the toxicity of titanium dioxide (TiO_2_)nanoparticles on L929 fibroblasts. It was shown that impedance-based monitoring is a valuable tool to investigate the cytotoxicity of nanoparticles [20]. To this end, there are no data available regarding the real-time effects of relevant endoprosthetic wear particles on cell viability in human cell cultures. To establish an impedance-based assay with metallic and ceramic wear particles, we used human primary fibroblasts based on their biological relevance and suitability for impedance-based monitoring and darkfield microscopy [20,21]. Moreover, since fibroblasts are also involved in particle-induced osteolytic events [15], we analyzed the gene expression of relevant chemokines and cytokines after exposure to metallic and ceramic wear particles.

## 2. Results

### 2.1. Impedance-Based Monitoring of Cell Viability

**Figure 1:**
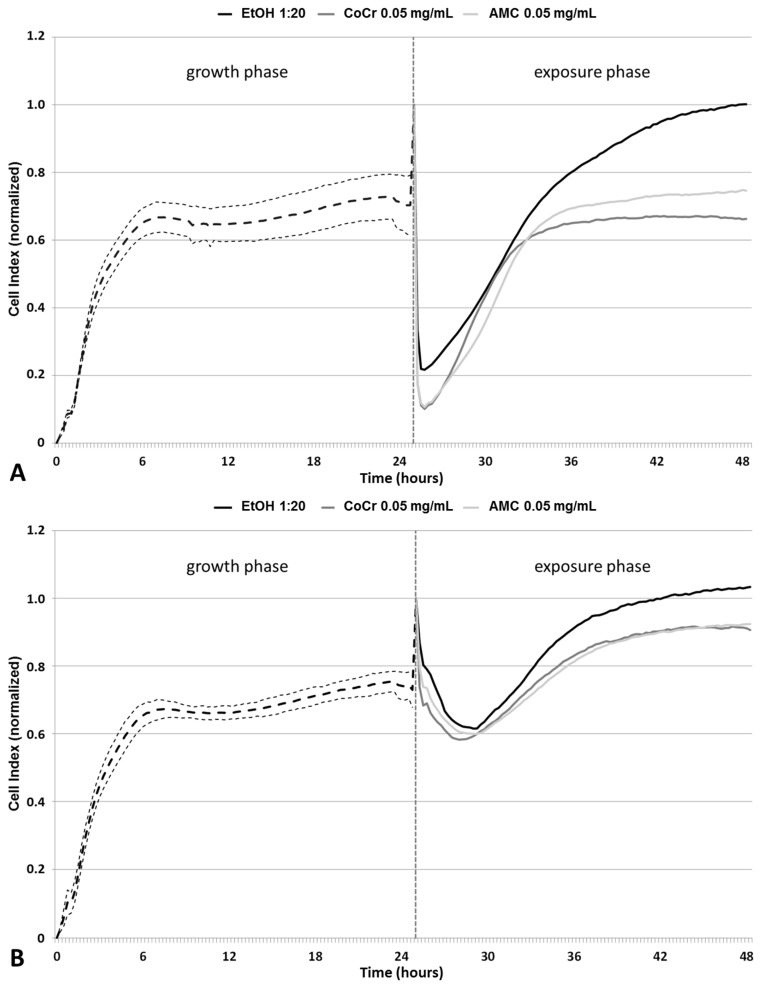
Impedance-based monitoring of human primary fibroblasts using the XCelligence Real Time Cell Analyzer. After 24 h of seeding, cells were exposed for another 24 h to (**A**) 0.05 mg/mL and (**B**) 0.01 mg/mL CoCr28Mo6 and AMC particles as well as the respective control. Impedance was monitored for another 24 h. Data were normalized to time point 25 h. For the growth phase, data are shown as mean value of the respective cell culture (thick dashed line) ± 95% confidence interval (fine dashed lines). Data of the exposure phase are shown as mean of three independent experiments performed in duplicates.

Impedance measurements of cells were carried out to determine cellular growth and viability during particle exposure (Figure 1). For this purpose, cells were seeded on gold-plated electrodes before an alternating current was applied. At lower frequencies the electrical impedance depends on the permeability of the cell membrane and small current variations due to environmental changes can be measured [22]. Viable adherent cells impede the electron flow, and the results are displayed as Cell Index (CI) [23], a unitless parameter, where CI = (impedance at time point *n*—impedance in the absence of cells)/nominal impedance value. A high CI indicates cell proliferation and thus viability, while a low CI indicates loss of cell adherence, cell death, and a reduction in the cell number. The increase of the CI during the first six hours after cell seeding indicated an increased adherence and cellular proliferation, after which a plateau was reached. Afterwards, a constant CI (around 0.7 in average) was detectable for all cell cultures. After 25 h of incubation, the cell culture media were replaced by either particle dispersions or control (1:20 *v*/*v* and 1:100 *v*/*v* EtOH, diluted in cell culture medium) solutions, which was marked by a peak in the CI due to temperature changes. For both controls, a time-dependent increase of viability was measured. The CI levels after 48 h were higher compared to CIs measured directly before media exchange (Figure 1A,B).

Exposure to CoCr28Mo6 and AMC particles resulted in a concentration-dependent decrease in viability indicated by lower CIs. At a concentration of 0.05 mg/mL, AMC particles had a weaker effect on the reduction of the measured CI than particles made from CoCr28Mo6.

### 2.2. Metabolic Activity

**Figure 2:**
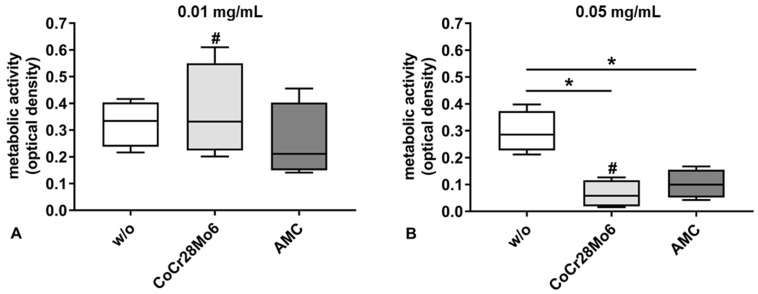
Cell viability of human primary fibroblasts after exposure to CoCr28Mo6 and AMC particles using concentrations of 0.01 mg/mL (**A**) and 0.05 mg/mL (**B**). Cells were transferred into standard culture plates allowing adherence over 24 h. Afterwards, the cell culture medium was replaced by different concentrations of particles and EtOH (control). After 48 h the metabolic activity was determined by the WST-1 assay. Data are shown as box plots (*n* = 4). Differences between groups were calculated with the Mann-Whitney-*U*-Test (* *p* < 0.05, treated vs. untreated; # *p* < 0.05, 0.05 mg/mL vs. 0.01 mg/mL).

The metabolic activity of cells was significantly reduced by the higher concentration (0.05 mg/mL) of particles (Figure 2B). In detail, exposure to CoCr28Mo6 and AMC particles at the concentration of 0.05 mg/mL resulted in significantly decreased cell metabolism compared to the respective control (both: *p* = 0.029). Incubation with the lower particle concentration (0.01 mg/mL) resulted in unchanged metabolic activity compared to control cells (Figure 2A). For CoCr28Mo6 treated cells, a significantly concentration-dependent effect was detectable (*p* = 0.029).

### 2.3. Expression of Osteolytic Mediators

**Figure 3:**
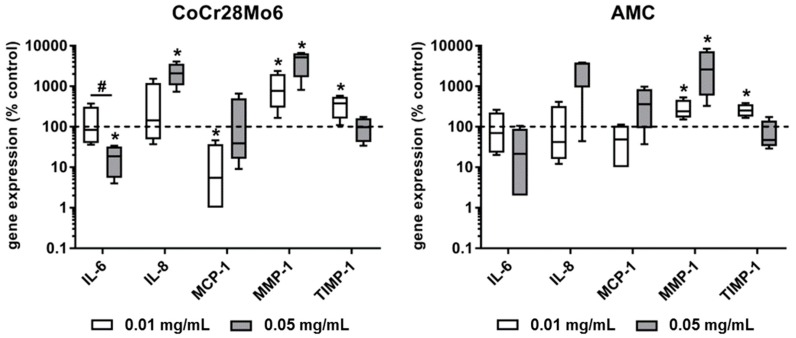
Gene expression analyses of pro-osteolytic markers in human primary fibroblasts following exposure to CoCr28Mo6 and AMC particles. Cells were cultivated in standard cell culture plates. After 24 h of adherence cells were exposed to particles over a period of 96 h. Afterwards, total RNA was isolated and reverse transcribed. Gene expression analyses were performed by qRT-PCR. Data are presented as box plots (*n* = 4; all data are in relation to unstimulated cells (%)). Significance was calculated using the Mann-Whitney-*U*-test (* *p* < 0.05, stimulated vs. unstimulated; # *p* < 0.05, 0.01 mg/mL vs. 0.05 mg/mL).

Exposure of fibroblasts to both types of particles resulted in a concentration-dependent decrease of mRNA levels for *IL-6*. This effect was significant (*p* = 0.029) for CoCr28Mo6 particles. *IL-8* expression levels were induced after exposure to the higher concentration of metallic and ceramic wear particles; however, a level of significance was only reached after treatment with CoCr28Mo6 particles. Metallic particles at the lower concentration significantly reduced the *MCP-1* mRNA levels (*p* = 0.029). For *MMP-1*, a significant upregulation of gene expression rates was determined in human fibroblasts after exposure to CoCr28Mo6 and AMC particles (all: *p* = 0.029). Here, a concentration-dependent tendency for *MMP-1* induction was observed. Comparing the effects of metallic and ceramic particles, exposure to AMC particles led to weaker *MMP-1* expression levels. Similar to *IL-6* expression rates, a reverse concentration-dependent effect on *TIMP-1* gene expression was detectable. The lower concentration of both CoCr28Mo6 and AMC particles induced a significant upregulation of *TIMP-1* in fibroblasts. Regarding the ratio of *MMP-1* to *TIMP-1*, a balanced ratio was shown after treatment with the lower concentration of AMC particles, whereas CoCr28Mo6 particles induced much higher *MMP-1* expression rates (Figure 3).

### 2.4. Determination of Particle Accumulation by Darkfield Microscopy

**Figure 4:**
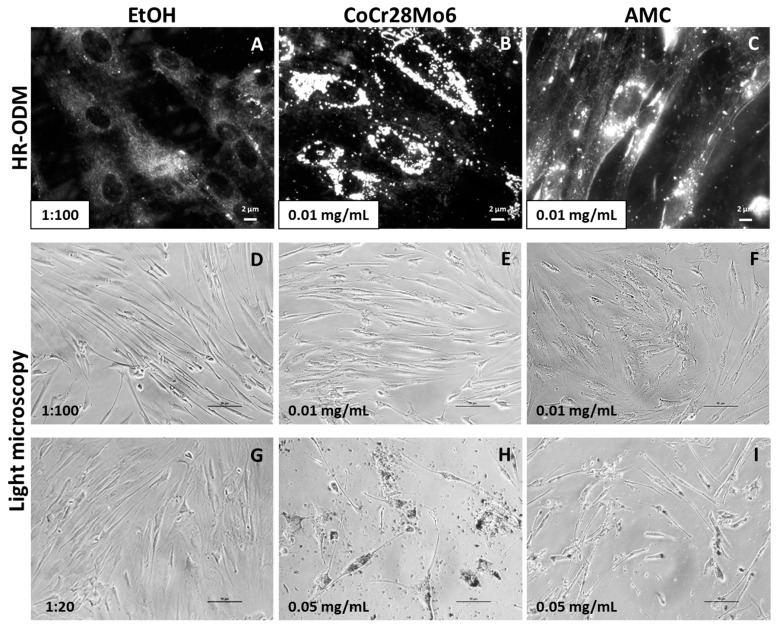
Representative pictures of human primary fibroblasts exposed to CoCr28Mo6 and AMC particles using HR-ODM (**A**–**C**; scale bar: 2 µm) and light microscopy (**D**–**I**; scale bar: 50 µm). Since particles were kept in EtOH to avoid agglomeration, control cell cultures were treated with EtOH in the respective concentration (**A**,**D**,**G**).

The presence of particles within cell cultures can be visualized by darkfield microscopy HR-ODM without labeling with fluorochromes. Thus, particle characteristics remain unchanged. Compared to untreated cells (Figure 4A), darkfield microscopy revealed bright shining particles mainly in the perinuclear region but not inside the nuclei (Figure 4B,C).

Using light microscopy, exposure to the lower particle concentration of 0.01 mg/mL did not appear to affect cellular morphology (Figure 4D–F). However, the higher concentration (0.05 mg/mL) had a visible effect on cell distribution and appearance (Figure 4H,I). In comparison to the EtOH control, cell confluency was reduced and fibroblast appearance was characterized by shorter cell length (Figure 4G). CoCr28Mo6 and AMC particles seemed to be ingested by cells.

### 2.5. Particle Detection by Field Emission Scanning Electron Microscopy (FESEM) Techniques

**Figure 5:**
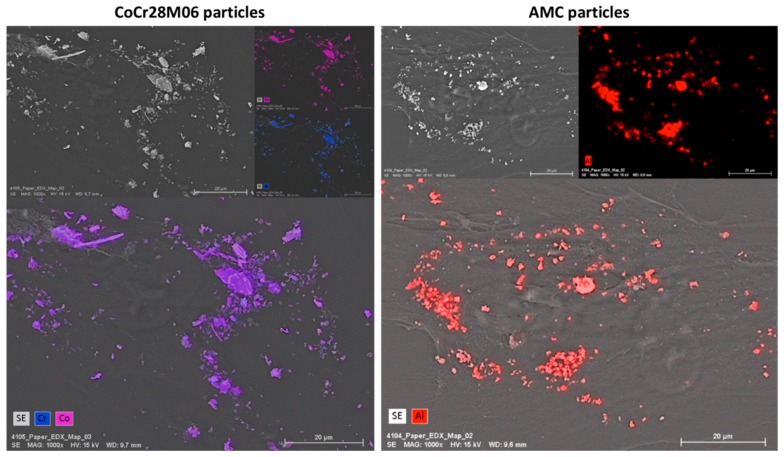
Representative FESEM pictures of human primary fibroblasts exposed to CoCr28Mo6 (**left panel**) and AMC (**right panel**) particles (scale bar: 20 µm). EDX measurements were carried out to identify the elements cobalt and chromium as well as aluminum.

FESEM and subsequent energy dispersive X-ray (EDX) analyses demonstrated the existence of the respective particle type on the cells. A contamination with other elements was not detectable, since exclusively the elements Co, Cr and Al were detected by EDX measurements. However, using EDX, it was not possible to distinguish between particles located on the cell surface or particles which were internalized by the cell (Figure 5).

**Figure 6:**
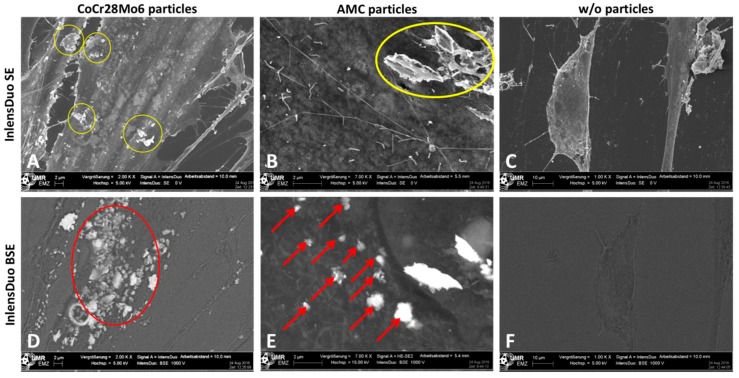
Human primary fibroblasts exposed to CoCr28Mo6 (**A**,**D**; scale bar: 2 µm) and AMC (**B**,**E**; scale bar: 2 µm) particles in comparison to unexposed cells (**C**,**F**; bar: 10 µm) analyzed with different Inlens Duo detector settings at 5 kV accelerating voltage. (**A**–**C**) Using the Inlens Duo SE detector, particles on the upper surface of the cell membrane were clearly visible (yellow circles). (**D**–**F**) Using the Inlens Duo BSE detector particles in the deeper region under the cell membrane were clearly visible (red arrows/circle). (**C**,**F**) No particles were detected by both InlensDuo detectors.

In order to receive information on whether particles were located on the surface of the outer cell membrane or inside the cell, different settings of the Inlens Duo detector were used. In the first setting, the Inlens Duo SE (secondary electrons) was used to get signals from the upper surface of the cell membrane. The visible particles were located on the surface of the outer cell membrane (Figure 6A,B). By using the Inlens Duo BSE (back scattered electrons), signals of regions deeper inside the cell were determined. The particles detected in addition to those visualized by the Inlens Duo SE were clearly located inside the cell (Figure 6D,E). The corresponding control experiments with non-exposed cells showed no particles on or in the cells (Figure 6C,F).

## 3. Discussion

The aim of this study was to assess the cellular uptake and cytotoxicity of metallic and ceramic wear particles using label-free methods and WST-1. We treated human primary fibroblasts with particles derived from a CoCr28Mo6 alloy and an alumina matrix composite (AMC) in two different concentrations (0.01 and 0.05 mg/mL). The biological relevance of these particle concentrations was shown in previous studies [18,19,24,25]. According to the manufacturer’s specifications, these particles were 500 nm in size. However, hydrodynamic diameters of 1.21 µm for AMC and 1.69 µm for CoCr28Mo6 were detected by dynamic light scattering (DLS), most probably due to particle agglomeration [19].

### 3.1. Determination of Cellular Viability by Impedance-Based Monitoring

The cytotoxicity of the particles was assessed by impedance-based monitoring of human fibroblasts using the XCelligence system. An important advantage of this system is the continuous monitoring of cellular behavior over a specific time period instead of traditional end-point measurements [26,27]. Therefore, differences in cellular proliferation, attachment, and detachment can be determined during exposure, as shown by Cimpan et al. (2013) [20], who used the impedance-based sensing approach to investigate the effects of various types of TiO_2_ nanoparticles on fibroblast viability and proliferation. The results from impedance measurements were mostly in line with Trypan Blue exclusion tests and HR-ODM, proving that this set-up is a suitable tool to assess nanoparticle toxicity [20]. In our present study, we exposed human fibroblasts to clinically-relevant metallic and ceramic wear particles, and monitored cellular proliferation and adhesion. Similar to the observations of Cimpan et al. [20], cells seeded onto the surface of the wells in the E-plates entered a proliferation phase during the first six to eight hours. This was indicated by a high increase in measured CI. Afterwards, the curve reached a plateau reflecting the stationary phase where cells stopped proliferation due to limited space and/or lack of fresh cell culture medium. Compared to the controls, a concentration-dependent decrease in CI due to cellular detachment of cells and decreased proliferation was observed following exposure to the particles. In this context, it can be assumed that those cells were dying, most probably by either apoptosis or necrosis. The higher particle dose influenced cellular viability to a greater extent. This was supported by the significantly reduced cell metabolism in the WST-1 assay and by the light microscopy observations, which showed a decrease in the cell number and changes in cellular morphology indicative of apoptosis. Morphological changes due to particle number and chemical composition were visible by light microscopy. Exposure to AMC particles did not alter cell morphology to the same extent as CoCr28Mo6 particles. Fibroblasts treated with CoCr28Mo6 particles at the higher concentration displayed more swelling than those exposed to AMC particles. We assumed that fibroblasts ingested more metallic particles, which, in turn, had higher cytotoxic effects, resulting in reduced viability. Although our data revealed that material composition and particle concentration have an impact on cellular survival, more systematic studies are necessary to point out the correlation of particle physico-chemical characteristics and cytotoxic outcome.

The greater impact of CoCr28Mo6 particles on fibroblast survival rates could also be explained by the additional release of ions. In a previous study, we have shown that cobalt and chromium ions were able to induce necrosis in human osteoblasts and adherent peripheral blood monocytic cells (PBMCs) [28]. Moreover, we could show that exposure of human osteoblasts to CoCr28Mo6 particles resulted in altered cell morphology and decreased metabolism [19]. However, in further studies, the impact of particle and ion treatment should be directly compared by impedance-based monitoring to point out specific differences on cell viability.

Our results suggest that impedance-based monitoring of cell viability during particle exposure could be a helpful tool to analyze the impact of wear particles. The observations were validated by end-point measurements of cell metabolism, as well as light microscopy. In future studies, impedance-based monitoring should also be carried out to investigate the cytotoxic effects of endoprosthetic wear particles on human osteoblasts and macrophages. For the latter, it is well known that this cell population is highly important for cell-particle interactions [6], and impedance-based monitoring may give new insights in the pathological process of particle disease at the cellular level.

### 3.2. Induction of Pro-Osteolytic Mediators

Wear particles have the ability to induce the secretion of different cytokines, such as IL-6, IL-1β, or TNF-α in monocytes/macrophages, lymphocytes, osteoblasts, as well as fibroblasts [29]. In previous studies, we analyzed the biological effects of wear particles and corrosion products in all the mentioned cell types except for fibroblasts [19,28]. Many fibroblasts can be found in the interfacial membrane of patients with particle-induced osteolysis, where they are supposed to be the major cell type for RANKL production [30]. In the presence of wear particles, fibroblasts produce additional pro-inflammatory cytokines and chemokines which maintain inflammation and contribute to the recruitment of osteoclast precursor cells [30]. Our data revealed that particles derived from a CoCr28Mo6 alloy induced *IL-8* gene expression at the higher concentration, whereas *IL-6* expression rates were reduced. In a previous study, analyzing gene expression, as well as protein levels of IL-6 and *IL-8* in human osteoblasts and PBMC [19], we showed similar results of *IL-6* and *IL-8* gene expression rates in PBMCs; however, in osteoblastic cells, we were unable to measure gene expression of both cytokines. Although a level of significance was not reached after exposure to AMC particles due to high inter-individual differences in fibroblastic cultures, a comparable tendency for increased levels of *IL-8* mRNA after exposure to the higher particle concentration was found. Our data demonstrated that particles from CoCr28Mo6 and AMC can induce relevant pro-osteolytic mediators on mRNA levels in fibroblasts; however, the translation and subsequent release of protein were not determined and this has to be investigated in further studies.

We also determined the gene expression rates of *MMP-1* and its natural inhibitor *TIMP-1* to analyze the extent of possible matrix degradation processes by fibroblasts. We were able to detect significantly upregulated gene expression rates of *MMP-1* for both particles at both particle concentrations. Interestingly, the lower particle concentration led to enhanced *TIMP-1* transcripts, whereas the higher concentration resulted in decreased ones. While a balanced *MMP-1/TIMP-1* ratio was determined when cells were treated with the lower dose of AMC particles (0.01 mg/mL), the increased *MMP-1* gene expression levels and the concomitant decreased TIMP-1 expression at the higher particle concentration indicated a massive induction of bone-degrading effects. However, in further studies, it has to be determined whether upregulated gene expression levels correspond with enhanced levels of active protein. Regarding this aspect, we have already shown that both particle types did not induce the synthesis rate of active MMP-1 protein in human osteoblasts, despite highly-induced gene expression rates [19].

### 3.3. Determination of Particle Ingestion

It is generally known that fibroblasts are able to phagocytise wear particles, and thus, mediating osteolytic events in the periprosthetic tissue [31]. It can be assumed that particle uptake into fibroblasts depends on particle characteristics, among which are size, morphology, and chemical composition. In this context, the study of Allouni et al. (2012) demonstrated a relationship between the size and shape of TiO_2_ nanoparticles and particle uptake in fibroblasts [21]. In our study, light microscopy as well as darkfield microscopy indicated ingested CoCr28Mo6 and AMC particles in human fibroblasts. Using darkfield microscopy, it could be assumed that the brighter shining particles were located on the outer membrane, whereas darker ones were located within the cells. However, the mechanism of particle ingestion into cells was not the objective of this study. To this end, we were able to visualize CoCr28Mo6 and AMC particles in human cell cultures by darkfield microscopy, which is a prerequisite for further testing. Using this methodological approach, it is possible to visualize particles within cell cultures without the need to label the particles, and thus, avoid modifying the particles characteristics and effects. Moreover, by using this setting, particle binding and uptake can be tracked in fixed cells or by live cell imaging [32,33]. Therefore, we intend to determine the cell-to-particle interactions by CytoViva™ darkfield microscopy [21] in further studies.

To validate whether particles were located above or under the cell membrane, we used a special setting of the FESEM. Compared to the transmission electron microscopy (TEM) technique, the advantage of the FESEM technique is the easy handling and sample preparation. Preparation of ultrathin sections (<100 nm) for TEM is difficult because there are embedded particles with dimensions that are larger than the section thickness. The FESEM technique, together with the element distribution data of the EDX mapping, proved the presence of ingested particles under the cell membrane; however, we were not able to validate the specific location of particles within cell structures, as shown in other studies [16,17,21,34].

## 4. Materials and Methods

### 4.1. Isolation of Human Fibroblasts

Human fibroblasts were isolated from human eyelid or breast skin biopsies after receiving approval from the ethical committee of the University of Rostock (registration No. A 2013-0092, approved on 10 August 2013). After removing excessive adipose tissue from the skin biopsies, the tissue was cut into small segments of 2–3 mm edge to edge lengths. Afterwards, 2 to 3 tissue sections were transferred into 6-well cell culture plates with the epidermal layer upwards. Air drying of the surface was done over 20 min with subsequent incubation in Dulbecco’s modified Eagle Medium (DMEM, Gibco Invitrogen, Darmstadt, Germany) containing 10% fetal calf serum (FCS), 1% penicillin/streptomycin, 1% amphotericin B (all: Gibco Invitrogen) under standard culture conditions (humidified atmosphere of 37 °C, 5% CO_2_). After reaching confluence, cells were transferred into a 75 cm^2^ cell culture flask. The cell culture media was changed every second day.

### 4.2. Particle Exposure

**Figure 7:**
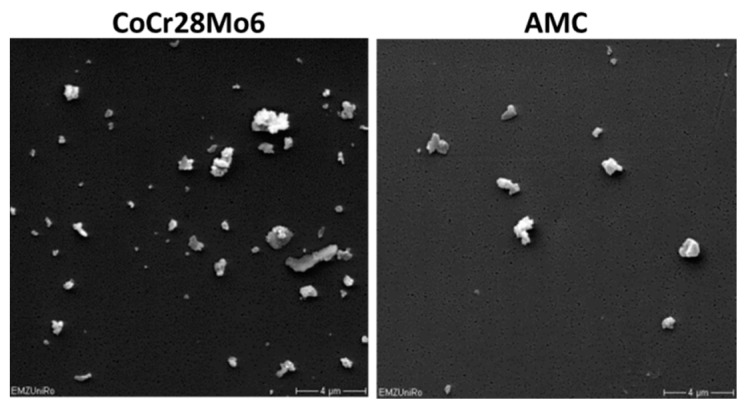
Representative FESEM pictures of CoCr28Mo6 and AMC particles (scale bar: 4 µm).

Particles derived from an alumina matrix composite (AMC), as well as from a cobalt-chromium-molybdenum alloy (CoCr28Mo6), were purchased from Continuum Blue (Cardiff, UK) (Figure 7). According to the manufacturer, the particles were guaranteed to be endotoxin free. Particle characteristics are described in Table 1. The mean size of dry particles was 500 nm for either particle type (manufacturer’s specification). To determine the diameter of particles in the stock solution containing 70% ethanol (EtOH), a volume of 250 µL was filtered through polycarbonate filters (Pieper Filter GmbH, Bad Zwischenahn, Germany) with 0.05 µm pore size. Analysis of the respective size of particles on the filter was carried out by scanning electron microscopy (MERLIN VP Compact VP, Carl Zeiss, Oberhausen, Germany) and subsequent Leica QWin image analysis software (Leica Microsystems GmbH, Wetzlar, Germany). Additionally, the agglomerate mean sizes of particles in aqueous solution were determined by dynamic light scattering (DLS, Zetasizer NS, Malvern Instruments, Malvern, UK) measurements, and are listed in Table 1. Data indicated agglomerated particles in the respective particle solutions.

A particle stock solution of 1 mg/mL was prepared in 70% ethanol (EtOH) to reduce particle agglomeration. For cell experiments, the particle stock solutions were homogenized by ultrasonication (UP100H, Hielscher Ultrasonics GmbH, Teltow, Germany) for 2 min at an amplitude of 80% and a cycle of 0.4. The preparation of the particle dilutions was done in complete cell culture medium (DMEM). Additionally, control solutions of DMEM and the respective dilution of ethanol (70%) were prepared. The morphology of particle-exposed fibroblasts was documented by light microscopy using a magnification of 200× (Nikon ECLIPSE TS100, Nikon GmbH, Duesseldorf, Germany).

### 4.3. Determination of Cell Viability and Toxicity

For impedance-based cell monitoring, the XCelligence Real Time Cell Analyzer Dual Plate (ACEA Biosciences Inc., San Diego, CA, USA) was used. This system is able to measure the electrical impedance across interdigitated microelectrodes which are situated at the bottom of cell culture wells [20]. By cell cultivation onto electrodes, impedance increases as the number of cells increases. The impedance measurement is given as a dimensionless Cell Index (CI), and reflects cell status, morphology, adherence, and viability. A high CI means less cytotoxicity because of a high number of adherent cells and a low CI means a loss of cell adherence and cell number.

To monitor the influence of CoCr28Mo6 and AMC particles, 10,000 cells per well were seeded in 16-well E-plates. After 30 min at room temperature, the E-plates were placed in an incubator at 37 °C and 5% CO_2_. After 24 h, the medium was replaced with particle-containing medium, and impedance was measured over 24 h. The cell growth, expressed as CI curve, was monitored for each well over the whole 48 h period. Particle concentrations of 0.05 mg/mL and 0.01 mg/mL were used. Cells cultured in DMEM alone and DMEM containing 70% EtOH in the respective dilutions (1:20, 1:100) served as controls. To assess the impedance of particles in the medium, complete DMEM with particles (0.05 mg/mL and 0.01 mg/mL) was used. The measurements were done under standard culture conditions.

The CI values of the control group (EtOH) were compensated for by the CI values of medium only, while the particle-exposed groups were adjusted for the CI values of medium plus particles. The following subtractions at each time-point were performed:

CI (control cells + EtOH) = CI (control cells + medium + EtOH) − CI (medium without EtOH)(1)

CI (treated cells) = CI (treated cells + medium + EtOH + particles) − CI (medium + EtOH + particles)(2)

The compensated CIs were then normalized by the CI at 25 h which was the time-point of cell exposure to particles. The normalized CI was calculated as follows:

CI (normalized) = CI (specific time-point)/CI (normalization time-point)(3)

Besides impedance measurements, the cell viability of human fibroblasts was determined by the end-point assay WST-1 (Roche, Penzberg, Germany), using 30,000 human fibroblasts per well in 24-well plates. After 24 h under standard cell culture conditions, cells were exposed to CoCr28Mo6 and AMC particles at 0.05 mg/mL and 0.01 mg/mL. Cells treated with the respective concentration of 70% EtOH served as controls. After 48 h of incubation, the particle/EtOH solutions were removed and cells were incubated with a defined volume of WST-1/medium reagent (ratio 1:10) at 37 °C and 5% CO_2_ for 30 min. Afterwards, supernatants were transferred into 96-well cell culture plates (in duplicates) to determine the absorption at 450 nm (reference wave length: 630 nm) in a microplate reader (Dynex Technologies, Denkendorf, Germany).

### 4.4. Gene Expression Analyses

To analyze the gene expression rates of pro-inflammatoric mediators, the RNA of treated and untreated cells was isolated using the Direct-zol kit (Zymo Research, Freiburg, Germany) according to the instructions of the supplier. Then, 50 ng of total RNA was reverse transcribed using the High Capacity cDNA Kit (Applied Biosystems, Forster City, CA, USA) as described by the manufacturer. Relative quantification of target cDNA (IL-6, IL-8, MCP-1, MMP-1 and TIMP-1) was performed by quantitative real-time PCR (qRT-PCR, qTower 2.0, Analytik Jena, Jena, Germany) using the innuMix qPCR MasterMix SyGreen (Analytik Jena) and the primers (Sigma-Aldrich, St. Louis, MO, USA) described in Jonitz-Heincke et al. [26]. QRT-PCR analysis was performed under the following conditions: 2 min at 95 °C, 40 cycles of 95 °C (5 s) and 65 °C (25 s). The reactions were performed in duplicate. The relative expression of each mRNA compared with the housekeeper gene HPRT was calculated by the equation ∆*C*_t_ = *C*t_target_ − *C*t_HPRT_. The relative amount of target mRNA in the unstimulated cells and treated cells was expressed as 2-(∆∆*C*_t_), where ∆∆*C*t_treatment_ = *C*t_target_ − *C*t_control_.

### 4.5. High-Resolution Optical Darkfield Microscopy (HR-ODM)

For particle visualization in cell cultures, a customized condenser with ultrahigh resolution (CytoViva^®^ 130, Auburn, AL, USA) connected to a research grade optical microscope (Olympus, BX41, Tokyo, Japan) and a powerful light source (EXFO120 PC, Photonic Solution Inc., Mississauga, ON, Canada) were used. For this instance, fibroblasts were seeded in cell culture chamber slides, allowing adherence over a period of 24 h. Afterwards, cells were exposed to CoCr28Mo6 and AMC particles in a concentration of 0.01 mg/mL over a period of 24 h. Cells were fixed with paraformaldehyde (PFA) for 15 min at room temperature. After fixation, the cells were washed twice with PBS and once with sterile water; then, the chambers were removed from the slides. A drop of Eukitt mounting medium (VWR International LLC., Oslo, Norway) was added to the slides and encased with a cover-slip. The fixed cells were visualized with a 100× oil immersion objective. The digital images were captured with a cooled camera (model XLMCT, Dage-MTI, Michigan City, IN, USA) and DageXponent software (version 1.3, Dage-MTI, Michigan City, IN, USA).

### 4.6. Field Emission Scanning Electron Microscopy (FESEM) and Elementary Analysis (EDX)

Human fibroblasts (20,000 cells) were seeded on Thermanox^®^ cover slips (ThermoFisher, Waltham, MA, USA). After 24 h, cells were exposed to CoCr28Mo6 and AMC particles (0.01 mg/mL) over a period of 24 h. Afterwards, cells were fixed paraformaldehyde (PFA, 25% glutaraldehyde, 0.4 mol/L phosphate buffer, ad 100 mL Aqua dest.) at 4 °C. After washing with sodium phosphate buffer, cells were dehydrated with acetone, followed by critical point drying (K850, Quorum Technologies Ltd., East Sussex, UK). The cover slips were fixed on sample holders (alumina) for further coating with a thin carbon film. Pictures were taken with a field emission scanning electron microscope (MERLIN VP Compact VP, Carl Zeiss, Oberhausen, Germany) using an Inlens Duo Detector. The acceleration voltage was 5 kV. Additionally, element analysis using energy dispersive X-ray measurement (EDX, Quantax QX400, Bruker, Billerica, MA, USA) was done.

### 4.7. Data Illustration and Statistical Analysis

If not otherwise stated, data are presented as box plots. Boxes denote interquartile ranges, horizontal lines within the boxes denote medians, and whiskers denote minimum and maximum values. For all analyses, the cultures (in duplicates) of human fibroblasts from a minimum of three independent donors were used. Since the data obtained were not normally distributed, statistical differences between two datasets were calculated with the Mann-Whitney-*U*-test using SPSS 20 (IBM Deutschland, Ehningen, Germany). The level of significance was set to a *p*-value less than 0.05 (*p* < 0.05).

## 5. Conclusions

In conclusion, the label-free methodological approaches ensured an interference-free evaluation of the cytotoxic effects of two particle types. The impedance-based real-time monitoring provides detailed insights on the dynamic cellular viability and proliferation during exposure to metallic and ceramic wear particles compared to end-point measurements. Particle exposure affected cell viability in a concentration-dependent manner, and the effects were more pronounced when using the particles from a CoCr28Mo6 alloy compared to AMC particles. The outcome of the measurements was in accordance with our light microscopy observations, the determined cellular metabolism, as well as the enhanced gene expression rates of cytokines and chemokines. It can be concluded that the impedance-based approach is a valuable tool for testing particle cytotoxicity. In order to analyze particle uptake into cells, we conducted high-resolution darkfield microscopy as well as performing FESEM techniques. Both microscopic approaches revealed the presence of metallic and ceramic particles within the cells.

## Figures and Tables

**Table 1:** Specifications of the used particles [19].

Characteristics	CoCr28Mo6	Alumina Matrix Composite (AMC)
Particle mean size (dry, manufacturer’s specification)	500 nm	500 nm
Equivalent diameter ([mean/min/max], in 70% EtOH, FESEM analyses)	200 nm (100 nm; 1000 nm)	300 nm (100 nm; 600 nm)
Agglomerate mean size (in aqueous solution, DLS measurements)	ZD: 1.69 µm PdI: 0.22	ZD: 1.21 µm PdI: 0.27
Particle morphology (according to ASTM-F1877-05)	flake-like to globular (cauliflower)	granular, irregular, angulated

Abbreviations: DLS: Dynamic light scattering; ASTM: American Society for Testing and Materials; ZD: z-Average Diameter; PdI: Polydispersity Index; EtOH: ethanol; FESEM: field emission scanning electron microscopy.

## References

[1] Sadoghi P., Liebensteiner M., Agreiter M., Leithner A., Bohler N., Labek G. (2013). Revision surgery after total joint arthroplasty: A complication-based analysis using worldwide arthroplasty registers. J. Arthroplasty.

[2] Jacobs J.J., Campbell P.A., Konttinen T. (2008). How has the biologic reaction to wear particles changed with newer bearing surfaces?. J. Am. Acad. Orthop. Surg..

[3] Amirhosseini M., Andersson G., Aspenberg P., Fahlgren A. (2017). Mechanical instability and titanium particles induce similar transcriptomic changes in a rat model for periprosthetic osteolysis and aseptic loosening. Bone Rep..

[4] Baumann B., Rader C.P., Wirtz D.C., Rader C.P., Reichel H. (2008). Partikelkrankheit. Revisionsendoprothetik der Hüftpfanne.

[5] Kadoya Y., Revell P.A., al-Saffar N., Kobayashi A., Scott G., Freeman M.A. (1996). Bone formation and bone resorption in failed total joint arthroplasties: Histomorphometric analysis with histochemical and immunohistochemical technique. J. Orthop. Res..

[6] Trindade M.C., Lind M., Sun D., Schurman D.J., Goodman S.B., Smith R.L. (2001). In vitro reaction to orthopaedic biomaterials by macrophages and lymphocytes isolated from patients undergoing revision surgery. Biomaterials.

[7] Ollivere B., Wimhurst J.A., Clark I.M., Donell S.T. (2012). Current concepts in osteolysis. J. Bone Joint Surg. Br..

[8] Hallab N.J., Jacobs J.J. (2009). Biologic effects of implant debris. Bull. NYU Hosp. Jt. Dis..

[9] Jacobs J.J., Roebuck K.A., Archibeck M., Hallab N.J., Glant T.T. (2001). Osteolysis: Basic science. Clin. Orthop. Relat. Res..

[10] Goodman S.B., Gibon E., Yao Z. (2013). The basic science of periprosthetic osteolysis. Instr. Course Lect..

[11] Gill H.S., Grammatopoulos G., Adshead S., Tsialogiannis E., Tsiridis E. (2012). Molecular and immune toxicity of CoCr nanoparticles in MoM hip arthroplasty. Trends Mol. Med..

[12] Potnis P.A., Dutta D.K., Wood S.C. (2013). Toll-like receptor 4 signaling pathway mediates proinflammatory immune response to cobalt-alloy particles. Cell Immunol..

[13] Pearl J.I., Ma T., Irani A.R., Huang Z., Robinson W.H., Smith R.L., Goodman S.B. (2011). Role of the Toll-like receptor pathway in the recognition of orthopedic implant wear-debris particles. Biomaterials.

[14] Nich C., Goodman S.B. (2014). Role of macrophages in the biological reaction to wear debris from joint replacements. J. Long Term. Eff. Med. Implants.

[15] Gu Q., Shi Q., Yang H. (2012). The role of TLR and chemokine in wear particle-induced aseptic loosening. J. Biomed. Biotechnol..

[16] Lohmann C.H., Schwartz Z., Koster G., Jahn U., Buchhorn G.H., MacDougall M.J., Casasola D., Liu Y., Sylvia V.L., Dean D.D. (2000). Phagocytosis of wear debris by osteoblasts affects differentiation and local factor production in a manner dependent on particle composition. Biomaterials.

[17] Lohmann C.H., Dean D.D., Koster G., Casasola D., Buchhorn G.H., Fink U., Schwartz Z., Boyan B.D. (2002). Ceramic and PMMA particles differentially affect osteoblast phenotype. Biomaterials.

[18] Lochner K., Fritsche A., Jonitz A., Hansmann D., Mueller P., Mueller-Hilke B., Bader R. (2011). The potential role of human osteoblasts for periprosthetic osteolysis following exposure to wear particles. Int. J. Mol. Med..

[19] Klinder A., Seyfarth A., Hansmann D., Bader R., Jonitz-Heincke A. (2018). Inflammatory response of human PBMCs and osteoblasts incubated with metallic and ceramic submicron particles. Front. Immunol..

[20] Cimpan M.R., Mordal T., Schölermann J., Allouni Z.E., Pliquett U., Cimpan E. (2013). An impedance-based high-throughput method for evaluating the cytotoxicity of nanoparticles. J. Phys. Conf. Ser..

[21] Allouni Z.E., Hol P.J., Cauqui M.A., Gjerdet N.R., Cimpan M.R. (2012). Role of physicochemical characteristics in the uptake of TiO_2_ nanoparticles by fibroblasts. Toxicol. In Vitro.

[22] Pliquett U. (2010). Bioimpedance: A review for food processing. Food Eng. Rev..

[23] Schoelermann J., Burtey A., Allouni Z.E., Gerdes H.H., Cimpan M.R. (2016). Contact-dependent transfer of TiO_2_ nanoparticles between mammalian cells. Nanotoxicology.

[24] Jonitz-Heincke A., Lochner K., Schulze C., Pohle D., Pustlauk W., Hansmann D., Bader R. (2016). Contribution of human osteoblasts and macrophages to bone matrix degradation and proinflammatory cytokine release after exposure to abrasive endoprosthetic wear particles. Mol. Med. Rep..

[25] Schulze C., Lochner K., Jonitz A., Lenz R., Duettmann O., Hansmann D., Bader R. (2013). Cell viability, collagen synthesis and cytokine expression in human osteoblasts following incubation with generated wear particles using different bone cements. Int. J. Mol. Med..

[26] Urcan E., Haertel U., Styllou M., Hickel R., Scherthan H., Reichl F.X. (2010). Real-time xCELLigence impedance analysis of the cytotoxicity of dental composite components on human gingival fibroblasts. Dent. Mater..

[27] Quereda J.J., Martinez-Alarcon L., Mendoca L., Majado M.J., Herrero-Medrano J.M., Pallares F.J., Rios A., Ramirez P., Munoz A., Ramis G. (2010). Validation of xCELLigence real-time cell analyzer to assess compatibility in xenotransplantation with pig-to-baboon model. Transplant. Proc..

[28] Jonitz-Heincke A., Tillmann J., Klinder A., Krueger S., Kretzer J.P., Hol P.J., Paulus A.C., Bader R. (2017). The impact of metal ion exposure on the cellular behavior of human osteoblasts and PBMCs: In vitro analyses of osteolytic processes. Materials.

[29] Wang J., Wang L., Fan Y. (2016). Adverse biological effect of TiO(2) and hydroxyapatite nanoparticles used in bone repair and replacement. Int. J. Mol. Sci..

[30] Purdue P.E., Koulouvaris P., Nestor B.J., Sculco T.P. (2006). The central role of wear debris in periprosthetic osteolysis. HSS J..

[31] Bitar D., Parvizi J. (2015). Biological response to prosthetic debris. World J. Orthop..

[32] Yang C., Uertz J., Yohan D., Chithrani B.D. (2014). Peptide modified gold nanoparticles for improved cellular uptake, nuclear transport, and intracellular retention. Nanoscale.

[33] Curry A.C., Crow M., Wax A. (2008). Molecular imaging of epidermal growth factor receptor in live cells with refractive index sensitivity using dark-field microspectroscopy and immunotargeted nanoparticles. J. Biomed. Opt..

[34] Faye P.A., Roualdes O., Rossignol F., Hartmann D.J., Desmouliere A. (2017). Engulfment of ceramic particles by fibroblasts does not alter cell behavior. Biomed. Mater..

